# Astrocyte and Neuronal Plasticity in the Somatosensory System

**DOI:** 10.1155/2015/732014

**Published:** 2015-08-04

**Authors:** Robert E. Sims, John B. Butcher, H. Rheinallt Parri, Stanislaw Glazewski

**Affiliations:** ^1^School of Life and Health Sciences, Aston University, Birmingham B4 7ET, UK; ^2^School of Life Sciences, Keele University, Keele ST5 5BG, UK

## Abstract

Changing the whisker complement on a rodent's snout can lead to two forms of experience-dependent plasticity (EDP) in the neurons of the barrel cortex, where whiskers are somatotopically represented. One form, termed coding plasticity, concerns changes in synaptic transmission and connectivity between neurons. This is thought to underlie learning and memory processes and so adaptation to a changing environment. The second, called homeostatic plasticity, serves to maintain a restricted dynamic range of neuronal activity thus preventing its saturation or total downregulation. Current explanatory models of cortical EDP are almost exclusively neurocentric. However, in recent years, increasing evidence has emerged on the role of astrocytes in brain function, including plasticity. Indeed, astrocytes appear as necessary partners of neurons at the core of the mechanisms of coding and homeostatic plasticity recorded in neurons. In addition to neuronal plasticity, several different forms of astrocytic plasticity have recently been discovered. They extend from changes in receptor expression and dynamic changes in morphology to alteration in gliotransmitter release. It is however unclear how astrocytic plasticity contributes to the neuronal EDP. Here, we review the known and possible roles for astrocytes in the barrel cortex, including its plasticity.

## 1. Introduction

Experience-dependent plasticity (EDP) is a fundamental property of the brain allowing neurons to refine patterns of connections during development, code and store information, and adjust activity of neurones in situations where their average activity is substantially reduced or increased. EDP, including its underlying mechanisms, has always received a great deal of attention as it is thought to be the mechanism for learning and retaining of life events hence also adaptation to continuously changing environment. A better understanding of the processes underlying EDP will not only enable us to shed light on such abilities but also better understand and possibly treat diseases such as Alzheimer's disease and brain traumas such as stroke, which are also linked to plasticity.

Of the many studies that have demonstrated EDP [1], one can generalise these into two general forms. Firstly, there is coding plasticity, which is always input-specific and concerns changes in synaptic transmission or connectivity of individual inputs to the neuron. Secondly, there is homeostatic plasticity, which commonly concerns changes in the global activity of neurones. While the major function of coding plasticity is to process specific information, homeostatic plasticity modifies excitatory and inhibitory inputs to neurones aiming to maintain a particular dynamic range of possible activity. This prevents synaptic saturation or total downregulation of neuronal activity [2].

## 2. The Barrel Cortex and Experience-Dependent Plasticity

**Figure 1:**
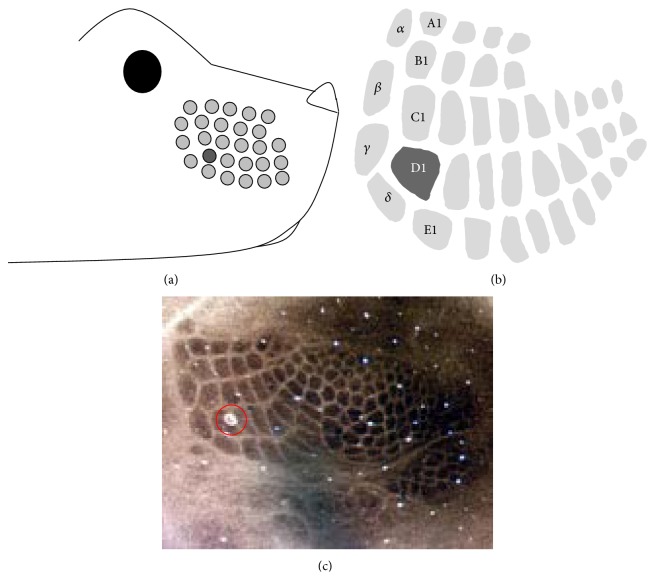
The murine whiskers have a corresponding somatotopic representation in the cortex. The location of the whiskers on the mouse's snout is shown in (a), with the corresponding barrels labelled in (b). The murine barrel field (cortical layer IV) was stained for cytochrome oxidase histochemistry (c). The lesion (red circle) was made by passing a small current through a carbon fibre electrode and indicates the D1 barrel. The somatotopic arrangement of barrels provides an excellent reference point for plasticity experiments by the removal of whiskers.

Rodents rely on several sensory systems to probe their immediate environment, but their whiskers to barrels system appears to be crucial [3], transmitting the sensory signal via several brainstem nuclei and the somatosensory thalamus to the contralateral primary somatosensory cortex and from here to other cortical and subcortical areas [3]. The pattern of the large whiskers on the muzzle of the two most studied rodents, rats and mice, is remarkably consistent and includes five rows. Each row contains several whiskers and there is an additional arc of whiskers, which is found between the 1st whiskers of each row containing four large whiskers giving a total of about 30 large whiskers on one side of the snout. This regularity of patterning and consistency between animals allows the specific identification of individual sensory whiskers (Figure 1). The pattern of whiskers on the rodent's snout is somatotopically represented along the whiskers to barrels system in the form of barrellettes in some of the nuclei of the somatosensory brainstem, barreloids in the somatosensory thalamus, and barrels in the somatosensory cortex (Figure 1). A barrel is a central element of the “barrel column” which spans all cortical layers and is a major recipient of the thalamic innervation [4]. Stimulus delivered to a particular whisker elicits neuronal firing, predominantly in that whisker's corresponding barrel column (principal input), but to a lesser extent also in the neurons of immediately surrounding whisker representations (surround inputs). The consistent somatotopic organisation of the cortical representation of whiskers, the substantial size of the barrel cortex, and its accessibility to electrophysiological recording coupled to the ease of inducing EDP by peripheral whisker manipulation have made the whisker-barrel cortex system the most widely studied model of cortical function and plasticity. Indeed, the physiological and molecular mechanisms underlying barrel cortex EDP focusing on changes in receptor expression and enzyme pathways and changes in synaptic strength in neurones have been examined in depth [3].

Changes in the experience of whiskers lead to plasticity of whisker-evoked barrel cortex responses to whisker stimulation. Barrel cortex EDP may be evoked via whisker deprivation, overstimulation, or Pavlovian (where an animal does not have an influence on the reinforcement) and instrumental conditioning (where an animal decides on reinforcement) involving whiskers [5–8].

## 3. Coding (Input-Dependent) Plasticity and the Barrel Cortex

**Figure 2:**
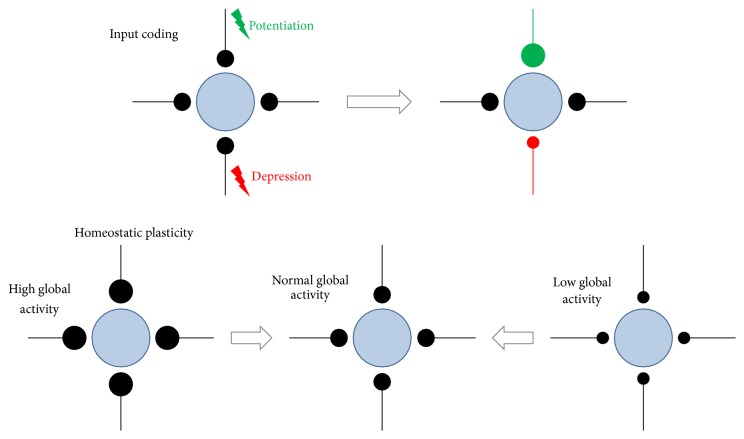
A schematic of coding and homeostatic plasticity. In coding plasticity, a specific pattern of activity on a particular input to the neuron leads to the strengthening or weakening of that connection. A common form of homeostatic plasticity known under the name of synaptic scaling involves proportional changes to all of the synapses to a neuron in order to keep its mean activity within a specified operating range. Blue circles represent postsynaptic cells and black lines/circles synaptic inputs. Wider synaptic inputs represent greater synaptic strength.

**Figure 3:**
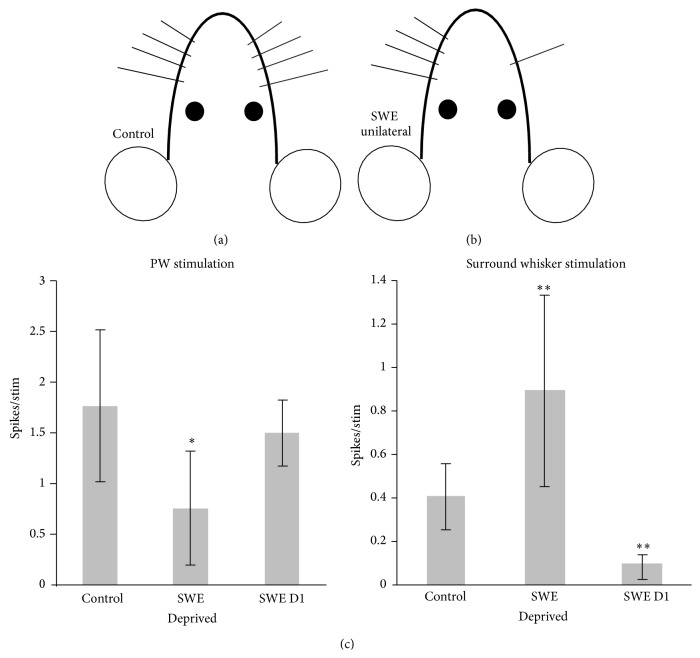
Plasticity in the barrel cortex can be induced by single-whisker experience (SWE). All whiskers except the D1 whisker were removed from one side of the face after P28 (a). Whiskers were kept deprived for 18 days followed by 5–8 days of deprived whisker regrowth, then whisker driven responses were recorded from the barrel cortex in anaesthetised mice. When whiskers were stimulated, recordings were taken from that whisker's corresponding principal whisker column (PW; b) and the immediately surrounding columns (c). In the principal whisker column, responses to stimulation of deprived whiskers but not the intact D1 whisker were significantly downregulated compared to those in the undeprived, control animals. However, surround responses driven by stimulation of the D1 whisker were significantly increased, while responses of the D1 column driven by the stimulation of deprived whiskers were significantly suppressed compared to undeprived, control animals (^*∗*^ *p* < 0.05, ^*∗∗*^ *p* < 0.01, Mann-Whitney *U*-test).

Coding plasticity (Figure 2) in the barrel cortex is often limited to early development and also to a particular cortical layer and specific experience [5, 9, 10]. This means that plasticity is regulated not only by the developmental neuronal age (critical period) but also by the induction method and possibly by previous experience [11, 12]. For example, in younger animals removing all but one whisker, known as single whisker experience (SWE), leads to lasting potentiation of the intact whisker-evoked responses, measured with increased number of action potentials per stimulus, in layers 2/3 of the barrel columns surrounding its own representation (and in some circumstances in its own representation) (Figure 3). Such a change in neuronal responsivity often occurs hours after deprivation onset [13–15]. Concurrently, principal whisker responses in deprived columns undergo suppression, which always precedes the development of potentiation [16]. In older animals potentiation of intact inputs is a principal form of plasticity [5, 6, 17, 18]. SWE elicits EDP in the barrel cortex, which does not have the time limit for expression except for layer IV barrels, where it can only be induced during the first few postnatal days. In contrast, removal of every second vibrissae termed “chessboard deprivation” removes all time and layer limits for plasticity.

In summary, input-specific plasticity in the barrel cortex always involves potentiation of the intact inputs and often the suppression of the deprived inputs and has critical periods, which depend on induction method [5, 6]. Interestingly, whisker-induced potentiation seems to depend on the same cellular and molecular mechanisms as long term potentiation (LTP) in the cortex [15, 19, 20].

## 4. Homeostatic Plasticity and the Barrel Cortex

A great deal of progress has been made in understanding the mechanisms of coding plasticity in the brain, but there is another form of plasticity, which has rather a house keeping than coding role, which is known as homeostatic plasticity. This form of plasticity limits excitatory drive and thereby prevents eventual excitotoxic damage during addition of excitatory connections during development [21–23]. Also, homeostatic plasticity accounts for precise balancing of LTD/LTP-like synaptic changes underlying memory codes to prevent synaptic saturation. From the several known mechanisms for homeostatic plasticity the most investigated is synaptic scaling, which is a negative feedback process that allows each cell to readjust the gain of synaptic input it receives based on how strongly the cell is excited. Calcium-dependent sensors are thought to play a role in this process by regulating the number of glutamate receptors at synapses [24], although other mechanisms may also exist for homeostatic plasticity. A good example of scaling was shown where excitatory neurones growing under a blockade of inhibition downscaled excitatory inputs while cells grown under a blockade of excitation upscaled their inputs [25]. As this form of synaptic scaling was found to be multiplicative or proportional, information stored as synaptic weights is preserved. Scaling was observed in variety of brain areas both* in vitro* and* in vivo* including the visual cortex where open eye potentiation, induced by monocular deprivation, is dependent on TNF-*α* and GluR1 [25–33]. Homeostatic upregulation of responses was also induced in the barrel cortex by the deprivation of all whiskers unilaterally [34]. It was found that the deprivation of all whiskers leads to an almost immediate downregulation of deprived inputs, which lasts for up to three days after deprivations onset. Initial suppression of deprived whisker responses is followed by a rebound in response magnitude that overshoots the original baseline response. This form of whisker deprivation induced homeostatic plasticity was not observed in TNF-*α* knock out animals, indicating a role for this cytokine. This is consistent with cell culture studies showing that TNF-*α* can upregulate the cell surface expression of AMPA receptors [27, 35].

## 5. Astrocytes

Astrocytes are a type of glial cell and are the most abundant cells found in the human brain. Their star-shaped bodies have many processes that surround synapses between neurons. In recent years, a picture has emerged whereby astrocytes have a far greater role in brain function than was previously envisaged. Once thought to provide a purely supportive role to neurons (sometimes thought of as gap fillers), they are now increasingly acknowledged as being active partners with neurons in synaptic communication in the brain. Consequently, it is recognised that a complete understanding of brain function requires an understanding of not only astrocyte function but also astrocyte-neuron interactions [36, 37].

## 6. Impact of Astrocytes on Neuronal Physiology

**Figure 4:**
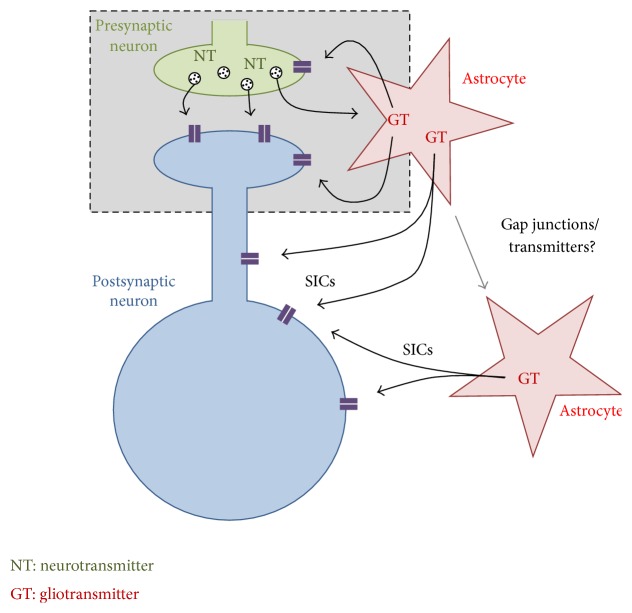
A schematic of astrocyte-neuronal signalling. The grey box represents the tripartite synapse system; astrocytes sense neurotransmitter release and respond with release of gliotransmitter at the synapse. Astrocytes also release glutamate to activate extrasynaptic NMDA receptors, which can undergo long term enhancement (LTE) with synaptic activity. Extrasynaptic release may derive from astrocytes ensheathing the synapse and possibly through recruitment of other astrocytes in a network. SICs: slow inward currents; NT: neurotransmitter; GT: gliotransmitter.

Whilst much remains to be discovered, there is growing evidence for an astrocytic role in neuronal synaptic plasticity and this role may prove to be crucial. The fact that astrocytes ensheath synapses and have close contact with pre- and postsynaptic elements gave rise to the tripartite synapse hypothesis [38] (Figure 4).

The central theme of the tripartite synapse is that astrocytes sense the same synaptic inputs as neurons and respond with intracellular Ca^2+^ elevations which in turn can elicit the release of gliotransmitters (GTs) such as ATP, D-serine, TNF-*α*, and glutamate. In the hippocampus, astrocytes release glutamate that acts presynaptically to increase release probability [39, 40]. Astrocytes in the hippocampus also mediate a form of heterosynaptic plasticity via release of ATP in response to synaptic activation and its degradation to adenosine [41]. The postsynaptic action of D-serine, released from astrocytes, is also necessary for the induction of long term potentiation (LTP) in the CA1 area [42], and in* in vivo* studies in the cortex it have found that activation of astrocytes by cholinergic afferents releases D-serine which is permissive for the induction of plasticity [43]. Evidence for a similar role for TNF*α* [39] indicates a role for astrocytes in metaplasticity, the ability to modulate the induction of synaptic plasticity [44]. Astrocytic glutamate has also been shown to facilitate the remodelling of somatosensory maps in the barrel cortex during the critical period [45].

Interestingly, astrocytic glutamate release can also activate extrasynaptic neuronal NMDA receptors, resulting in characteristic slow inward currents (SICs) [46, 47]. The first description of these currents in intact tissue was in the somatosensory thalamus [46]. SICs have since been recorded in many brain areas, including the cerebral cortex, and are recognised as one of the hallmarks of astrocyte-neuron signalling in the brain. The activation of NMDA receptors by astrocytic glutamate release can underlie synaptic strengthening [39, 48], but also the presence of SICs is a marker for astrocytic glutamate release which may also activate other pre- or postsynaptic glutamate receptor subtypes.

A major question facing neuroscientists is determining the role that recently revealed that astrocyte functions play a role in the mechanisms underlying adaptive animal behaviour such as EDP. The whisker-thalamus-barrel cortex system presents an ideal model for asking such questions and has already provided some intriguing clues.

## 7. Astrocyte Plasticity in the Somatosensory System

Neuronal plasticity is usually defined as changes which lead to an increase or decrease in the strength of synaptic signalling, associated with changes in neurotransmitter release or receptor expression. Because of the varied functions of astrocytes which may impact brain activity a broader definition of plasticity needs to be considered for these cells. Astrocytic plasticity therefore includes processes that regulate receptor and transporter expression, morphological plasticity, calcium signalling and oscillations, gliotransmitter release, and coupling via gap junctions. In general we are interested in astrocyte changes that ultimately affect neuronal function; therefore plasticity also applies to functions that may be classified as “housekeeping” such as processes that control ionic environment and metabolic demand homeostasis such as energy supply. Many examples of astrocytic plasticity are exhibited in the whisker-thalamus-barrel somatosensory system, and astrocytes have been proposed to be important in fundamental processes such as cortical sensory map organisation [49] and to interact with neuronal networks to enhance their computational properties [50]. Astrocyte plasticity could therefore have pivotal effects on somatosensory function. Since astrocytes are electrically nonexcitable but respond to different stimuli with elevations in intracellular calcium, there is great interest and focus on understanding the control of intracellular calcium and the calcium elevation mechanisms which are linked to specific physiological roles such as gliotransmitter release and haemodynamic control. At the astrocyte cellular level there is much to understand, with many transporters, exchangers, and ion channels being involved in the subcellular control of calcium and its release during signalling via a variety of pathways such as G protein coupled receptor activation releasing intracellular stores and cytoplasmic membrane channels such as TRP-A [48]. Understanding the mechanisms of these control mechanisms would lead to insight into how long term plasticity would affect astrocyte influenced roles.

## 8. Plasticity of Astrocyte Intracellular Signalling

There is longstanding and accumulating evidence that astrocyte Ca^2+^ activity can be modified. [51] demonstrated a sustained increase in astrocyte Ca^2+^ oscillation frequency following synaptic stimulation in hippocampal slices, indicative of an astrocyte functional modification following glutamate receptor activation. A similar increase by proposed adenosine A_2B_ receptor activation was also seen in cultured hippocampal slices [52] which was also seen by metabotropic glutamate and muscarinic receptor activation. Studies on the mechanism of such changes implicated increased receptor activity rather than intracellular signal cascade sensitivity changes [53]. The signalling calcium responsible for these elevations is due to release from intracellular stores. Metabotropic receptor-Gq G-protein activation and IP_3_ dependent release therefore appear to be the predominant pathway involved in the sensing of neuronal activity and neurotransmitter release by astrocytes.

In the somatosensory thalamus synaptically released glutamate acting at metabotropic glutamate (mGluR) group I receptors also resulted in an increased astrocytic oscillation frequency lasting over 1 hour [54] following inception. Together these results show that Gq-IP_3_ coupled neurotransmitter pathways can undergo signalling plasticity which affects cellular calcium elevation patterns for extended periods. As well as the somatosensory system, these pathways are a feature of numerous areas in the brain in which the activity can be instigated by stimulation of afferent sensory and intracerebral pathways. It is unclear at present what the function of these changes to calcium patterns is, and indeed the function of calcium elevations is a subject of much debate and not a little controversy. While studies involving the use of mouse models expressing alien G-protein coupled receptors and knockouts of IP_3_ signalling have suggested that this signalling pathway has no role in certain types of synaptic plasticity [55] other studies using the same models have shown astrocyte Ca^2+^ signalling involvement [56].

Calcium signals in astrocytes are far from homogenous and can display a range of temporal and spatial patterns, although local networks of astrocytes have been shown to exhibit local synchronous calcium waves [57]. Astrocytes can display calcium responses lasting 100s of milliseconds to tens of seconds [58, 59] and are confined to microdomains in astrocyte processes [60, 61]. These events are termed glissandi [62], propagating through large astrocyte populations and proposed to be involved in the haemodynamic response. So while the specific calcium activity patterns have not been assigned to particular physiological processes what is apparent is that many of the forms of astrocyte plasticity such as morphological changes, GT release, and gene expression are likely to involve calcium. There is also an association of calcium signal dysregulation with pathological states such as epilepsy [63, 64] where increased calcium elevation frequency was observed and also in models of Alzheimer's disease [65, 66]. It therefore seems that neurotransmitters and modulators released synaptically or changes in the local extracellular environment can induce long term plastic changes in astrocytic Ca^2+^ signalling, and this likely causes pathological changes in astrocytes that may contribute to disease states. These changes illustrate fundamental integration between synaptic activity and astrocyte response and activity.

One of the ways that changes in intracellular calcium signalling may be translated to functional brain plasticity is via the induction of morphological changes to astrocytes. Astrocytes possess a small soma with numerous processes that ramify into nebulous offshoots which surround synapses. Notably, astrocytes in the hypothalamus can undergo dramatic changes in morphology in response to hormonal changes during lactation in which process retraction increases synaptic glutamate spillover [67]. Whilst such changes are not apparent in the rest of the brain there is now evidence that morphological plasticity may be a widespread mechanism that affects synaptic activity. Imaging of processes shows that astrocyte processes exhibit dynamic spontaneous motility [68]. Studies on the mechanism of astrocyte morphology changes implicate mGluR activation and intracellular proteins such as pick 1, which are implicated in synaptic plasticity. Extended whisker stimulation has been found to increase synapse ensheathment by astrocyte processes in the barrel cortex [69].* In vivo* imaging using a cortical window of mouse barrel cortex also showed that astrocyte process plasticity correlates with sensory stimulation and that this is inhibited by mGluR group I inhibition and absent in IP_3_R_2_ knockout mice [70].

Perhaps because of the fact that most synaptic transmission to and within the brain is glutamatergic, most studies into neuron-astrocyte interactions and astrocyte plasticity changes implicate mGluRs. However, many neurotransmitters and neuromodulators are coupled via similar Gq-PLC-IP_3_ signalling pathways and so under different conditions many mediators may induce astrocyte changes and instigate plasticity. In this context it is interesting to consider the findings of Sun et al. [71] which showed that mGluRI expression in cortical astrocytes diminished during maturation while mGlurII expression prevailed. Such changes in expression may have implications for manifestations of synaptic plasticity in the somatosensory cortex, particularly those related to different developmental critical periods.

## 9. Plasticity of Astrocyte Networks

Astrocytes are coupled via gap junctions that allow the movement of ions and some signalling molecules between cells, because of this and the propagation of calcium waves the degree of coupling might underlie a functional population that modulates particular neuronal populations, and the extent of coupling may have functional consequences for neuronal activity. Indeed filling of astrocytes within layer 4 of the barrel cortex results in the filling of astrocytes in an oval pattern correlating to individual barrel boundaries. This is in contrast to nonbarrel areas where the resulting pattern was circular [72]. These experiments revealed that astrocytes within barrels are preferentially coupled to each other. These findings suggest that in barrel cortex the anatomical astrocyte arrangement is complementary to the anatomically defined neuronal structure. There is evidence from functional studies that such populations/networks have functional roles. Schipke et al. [73] found that this anatomical relationship was retained in functional responses since astrocytes within barrels responded preferentially to synaptic inputs to layer 4 rather than spontaneous activity generated in layer 2/3. This is in contrast to the situation in the VB thalamus where some astrocytes respond to either lemniscal (sensory) or corticothalamic afferents [74]. Further localised differences in astrocyte activity were seen in* in vivo* imaging from rodent somatosensory cortex [43]. Layer I astrocytes exhibited more spontaneous activity than layer 2/3 astrocytes and also displayed different patterns in their processes. Spontaneous activity patterns were independent of prevailing neuronal activity perhaps indicating inherently different regional roles.

Because of the interaction of astrocytes with synapses, the way they control transmitter uptake and release GT to modulate synaptic activity, astrocytic plasticity has the potential to change neuronal signalling and activity at a number of levels from the monosynaptic to local cellular network and large neuronal networks. For example, astrocytes have been shown to regulate UP states in* in vitro* recordings of S1 where the use of a calcium chelator in astrocytes inhibited the spontaneous and stimulated occurrence of UP states [75]. It is important to understand at which level or scale astrocytes relate to neurons and to what extent astrocytes behave in networks since this will ultimately determine the output effect on the behaviourally relevant neuronal network.

From the morphological, structural, and signalling characteristics of astrocytes it is clear that they have the potential to operate in a range of potentially different networks. To act in a network, astrocytes need to be able to transmit information to each other, and they have the potential to do this in at least two ways: they are structurally coupled via gap junctions; and they can also release GTs. Therefore astrocytes may act in radically different types of networks or the two types of signalling may act in concert.

## 10. Plasticity of Gliotransmitter Release

The ability of astrocytes to release amino acids in response to certain stimuli has been known for a considerable time [76], but over the last 20 years it has been found that such GT release can affect neighbouring synaptic and neuronal activity. As discussed above, some of the main GTs implicated in such modulation are d-serine, glutamate, ATP, adenosine, and GABA [77–79]. There is still considerable debate and controversy regarding the mechanisms of GT release, much centred on whether release is via calcium-dependent vesicular release, analogous to that seen in neurons [80, 81]. However, there are many other possible pathways for GT release such as stretch or ligand gated channels, transporters, or gap junction hemichannels. Some of which, such as the bestrophin channels may also be calcium-dependent [82].

Understanding the mechanism of GT release is important in order to enable the modulation of release and determine the physiological function of each GT. Many tools are indeed available for this in addition to pharmacological tools; specific transgenic animal models have been developed such as inhibition of SNARE dependent vesicular release, via dnSNARE mice [77] or selective tetanus toxin expression [83]. An IP_3_R_2_ knockout which is a knockout of the IP_3_ subtype expressed in astrocytes is also available in which spontaneous and Gq-protein mediated responses are inhibited [84]. Additionally, mice missing an astroglial connexin (Cx30) showed a lack of plasticity when depriving olfactory glomeruli in early development (P20) [85], while Cx43 KO mice were shown to lack potentiation of barrel responses when driven with high-frequency whisker stimulation [86]. Despite studies using such models providing data supporting astrocyte GT roles [56], there is still continuing debate on their specificity and how important GT release is to brain function [55, 81, 87].

While some controversy remains concerning release mechanisms, there is ample evidence that phasic astrocyte glutamate release occurs in the brain and that this can impact neuronal activity, particularly in the somatosensory system. Astrocyte glutamate release is manifested as slow inward currents (SICs). First described in slice preparations from the somatosensory VB thalamus [46] they have since been described in many brain areas including hippocampus spinal cord and nucleus accumbens where, in line with the tripartite synapse hypothesis, they can be induced by acute synaptic afferent activity [88]. SICs also occur spontaneously usually at low frequencies of the order of every few minutes [89]. In the ventrobasal thalamus SICs predominantly target extrasynaptic NR2B subunit containing NMDA receptors [89] and the resulting neuronal depolarisations caused by SICs can lead to neuronal firing and excitation of adjacent neighbouring neurons lead to local neuronal synchronization [54] which is also reported in other brain areas [47, 88, 90].

**Figure 5:**
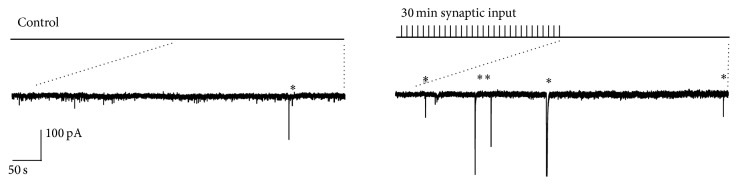
Long term enhancement (LTE) of astrocyte-derived slow inward currents (SICs). This representative example shows an increase in SICs recorded from thalamocortical neurons 60 minutes after 30 minutes of repetitive burst stimulation (10–20 stimulations at 50 Hz, every 5–10 seconds) of lemniscal and cortical afferents. Asterisks represent SICs.

An interesting finding in the VB thalamus that may point to important physiological roles of SICs and astrocyte glutamate release was that while synaptic stimulation in slice preparation elicited astrocytic Ca^2+^ elevations [89] there was no immediate SIC generation response. However, it was found that following extended periods of intermittent afferent input over 30 minutes (aimed at approximating physiological sensory input), SIC frequency was increased up to fourfold (Figure 5). The increase in frequency lasted for at least 1 hour following the cessation of the inducing synaptic activity and was termed “Long Term Enhancement” (LTE). This increase therefore indicates plasticity in the ability of astrocytes to spontaneously release glutamate. The induction of LTE plasticity was mediated by mGluR group I activation and the SICs following induction were inhibited by dialysing astrocytes with a Ca^2+^ chelator, indicating a Ca^2+^ dependent astrocytic glutamate release.

The physiological role of this plasticity remains to be determined; however since the VB thalamus is the nucleus that receives sensory whisker input it could be speculated that continual whisking by the animal would lead to such LTE plasticity* in vivo* and potentially contribute to neuronal activity and synchronisation.

This plasticity may not be confined to the thalamus and may be additionally involved in pathological states. Evidence from a pain model of hyperalgesia where recordings were conducted in dorsal horn neurones revealed an increase in SIC frequency [91]. This increase associated with increased activity in pain afferents could therefore represents an astrocyte GT release plasticity involvement in chronic pain. Very recently, the increased activity of astrocytes and glia has also been implicated in persistent pain in humans [92].

A number of groups have reported an increase in SIC frequency in epilepsy models [63, 93] and interestingly that mGluR_5_ mediated subsequent activation of NR2B containing NMDA receptors led to excitotoxicity [64]. In the APPswe model of Alzheimer's disease, an increase in SIC frequency has also been reported [66]. If an LTE type of plasticity is involved in pathological states it is not however clear if they underlie pathology or are a consequence of the condition which then contributes to functional deficit.

Although all cells produce TNF-*α*, the source of TNF-*α* relevant to homeostatic plasticity seems to be of glial origin [27]. Additionally, TNF-*α* is released by glia when neuronal activity levels are decreased [27]. Glial cells and especially astrocytes are in a good position to sense the general activity levels of cells because each envelops many neurons and so could provide a negative feedback signal to cells in their vicinity. According to this scenario, in the case of sensory deprivation, a drop in neuronal activity would be sensed by the glial cells, possibly resulting in the release of astrocytic release of TNF-*α*, which in turn would increase neuronal activity via the GluR1 upregulation or via another, unknown at present, mechanism. This last possibility is plausible as we did not detect synaptic scaling* ex vivo* in the barrel cortex of animals deprived of whiskers.

If TNF-*α* is released from astrocytes then the abolition of astrocytic calcium waves could therefore cause an increase in TNF-*α* levels and occlude the homeostatic effect of whisker deprivation. This is the testable hypothesis.

## 11. Astrocytes and Cortical Plasticity

It is becoming clear therefore that astrocytes can exhibit plasticity in various ways and at different anatomical network levels. There is also much known about the role of astrocytes in neuronal synaptic plasticity. While much of the foundational work on astrocyte roles in synaptic plasticity was conducted using the hippocampal slice model there is now increasing evidence that astrocytes are involved in synaptic plasticity in the barrel cortex and that this underlies changes in EDP.

An important finding is that astrocyte glutamate release is involved in spike timing dependent LTD. This form of plasticity is believed to be important in the formation of sensory map representations in the barrel cortex and so also occurs during the critical period. By stimulating L4 inputs to L2/3 pyramidal and eliciting coincident postsynaptic action potentials in conjunction with evoked EPSPs [94] showed that the resulting LTD was dependent on neuronal cannabinoid release acting at astrocyte CB1 receptors. CB1 activation caused astrocytic calcium increases which in turn led to vesicular glutamate release that targeted presynaptic NMDA receptors to cause synaptic depression.

An* in vivo* illustration of astrocyte roles in plasticity and their role indeed in gating synaptic plasticity was discovered by Takata et al. [43]. They recorded local field potential (LFP) responses in barrel cortex in response to air puff mechanical stimulation of rat whiskers. When whiskers were stimulated in this way at the same time as electrical stimulation of the cholinergic nucleus basalis of Meynert, the LFP response exhibited a sustained potentiation, indicating ACh induced synaptic plasticity. The potentiation was blocked by muscarinic antagonists and was not seen in IP_3_R_2_-KO mice, indicating the importance of ACh induced astrocyte calcium elevations. Astrocyte calcium signalling was accompanied by an increase in levels of the NMDAR coagonist D-serine. These experiments were conducted in rats older than 8 weeks showing a physiological role in plasticity in the barrel cortex beyond developmental critical periods. A previous observation [71] that the Gq-IP_3_ signalling coupled receptor mGluR5 was only expressed in young rodents prompted speculation that astrocyte calcium signalling pathways were not physiologically relevant in the adult. However,* in vivo* data [43] illustrates that this is not the case, at least for ascending cholinergic modulatory pathways.

The above examples illustrate astrocyte roles in input coding plasticity; however there is also emergent evidence of roles in homeostatic plasticity. Interestingly this indicates a role for the glial released cytokine TNF-*α*, which is also implicated in a number of disease states. The homeostatic cellular mechanism of action of TNF-*α* was demonstrated using cell and slice culture preparations [27]. This study proposed that astrocytes sense neuronal activity by responding to released glutamate and that this activation of astrocyte glutamate receptors inhibits TNF-*α* release. The study showed that TNF-*α* exposure caused an increase in neuronal synaptic AMPA receptor expression. The TNF-*α* effect could be mimicked by conditioned media from pure glial cultures but not from cultures which had transiently been exposed to glutamate to simulate neuronal activity. The conclusion therefore is that astrocytes constitutively release TNF-*α*, but with ongoing network activity and glutamate release this is inhibited. A reduction in network activity and glutamate leads to an increase in TNF-*α* induced AMPA receptor expression and an increase in synaptic strength, increasing the effect of excitation. This is therefore homeostatic synaptic plasticity.

## 12. Astrocyte Plasticity and Barrel Cortex EDP

Although astrocyte roles in barrel cortex synaptic plasticity now seem established, the possible interactions of astrocyte plasticity with these processes are unknown.

Recent findings showing astrocyte process plasticity in the hippocampus and barrel cortex [70] extend observations in the hypothalamus and indicate that astrocyte anatomical plasticity is a widespread feature. A medium to long term change on astrocyte synaptic coverage would be expected to have repercussions for input coding plasticity and possibly homeostatic plasticity.

The potentiation of inputs and so neuronal activity in undeprived barrels compared to deprived barrels would instill different metabolic requirements across the barrel cortex. From the descriptions of the dynamic nature of gap junction coupling in astrocyte networks [85, 95], an accompanying plasticity of astrocyte networks would be expected. The corresponding question of which changes would occur in neuronal networks as a result of instigating astrocyte network plasticity is unknown.

We have demonstrated a synaptically induced plasticity in astrocyte glutamate release in the rodent VB thalamus. The synaptic stimulation patterns were designed to approximate those produced by whisking patterns. It is known that the fidelity of sensory stimulation transmission differs between the thalamus and the cortex, and so it is not clear if the same afferent activity would induce astrocyte LTE in the barrel cortex. Astrocytes have been shown to be involved in input coding plasticity [43, 94] and their actions involve the activation of NMDA receptors. An extended increase in astrocyte glutamate release frequency would therefore be expected to interact with this form of plasticity, predictably increasing its effect or likelihood.

Astrocyte which released glutamate might also interact with homeostatic mechanisms. If astrocyte glutamate can act in an autocrine or paracrine manner then GT release plasticity would be expected to act at astrocyte receptors and suppress the release of TNF-*α* and inhibit homeostatic synaptic plasticity. To understand these possible interactions it is therefore important to understand the regulation of ongoing astrocyte activity and how changes in calcium oscillation frequency correlate to GT release. This understanding should also include a description of the roles of excitatory amino acid transporters which control ambient glutamate levels. It has recently been shown that astrocyte EAAT transporter mobility around the synapse is dynamically regulated [69] and that prolonged whisker stimulation leads to an increase in EAAT activity [69].

Astrocytic glutamate release has been implicated in the generation of cortical up states [75]. The elevated phasic release of GT following LTE would therefore be expected to increase the time spent in “UP” states and perhaps engender conditions that would promote the instigation of synaptic plasticity.

The emergence of new tools in recent years has made it an exciting time to be studying the role of astrocytes in the brain. Tools now exist which enable us to determine the cellular mechanisms of behavioural effects such as EDP* in vivo*. The IP_3_KO mouse is being extensively used to abrogate mechanisms that utilise astrocyte IP_3_ releasing pathways. The development of optogenetic tools that can be selectively expressed in neurons or astrocytes including one that activates channels or metabolic pathways provides great promise which will improve our understanding of the mechanisms of EDP.
